# Cytochrome P450 168A1 from *Pseudomonas aeruginosa* is involved in the hydroxylation of biologically relevant fatty acids

**DOI:** 10.1371/journal.pone.0265227

**Published:** 2022-03-21

**Authors:** Claire L. Price, Andrew G. S. Warrilow, Nicola J. Rolley, Josie E. Parker, Vera Thoss, Diane E. Kelly, Nicolae Corcionivoschi, Steven L. Kelly

**Affiliations:** 1 Centre for Cytochrome P450 Biodiversity, Institute of Life Science, Swansea University Medical School, Swansea University, Swansea, Wales, United Kingdom; 2 Plant Chemistry Group, School of Chemistry, Bangor University, Bangor, Gwynedd, Wales, United Kingdom; 3 Agri-Food and Biosciences Institute, Veterinary Science Division, Bacteriology Branch, Stoney Road, Stormont, Belfast, Northern Ireland, United Kingdom; 4 Faculty of Bioengineering of Animal Resources, Banat University of Agricultural Sciences and Veterinary Medicine, King Michael I of Romania, Timisoara, Romania; National Research Council, ITALY

## Abstract

The cytochrome P450 CYP168A1 from *Pseudomonas aeruginosa* was cloned and expressed in *Escherichia coli* followed by purification and characterization of function. CYP168A1 is a fatty acid hydroxylase that hydroxylates saturated fatty acids, including myristic (0.30 min^-1^), palmitic (1.61 min^-1^) and stearic acids (1.24 min^-1^), at both the ω-1- and ω-2-positions. However, CYP168A1 only hydroxylates unsaturated fatty acids, including palmitoleic (0.38 min^-1^), oleic (1.28 min^-1^) and linoleic acids (0.35 min^-1^), at the ω-1-position. CYP168A1 exhibited a catalytic preference for palmitic, oleic and stearic acids as substrates in keeping with the phosphatidylcholine-rich environment deep in the lung that is colonized by *P*. *aeruginosa*.

## Introduction

*Pseudomonas aeruginosa* is an opportunistic pathogen, and the leading cause of chronic lung infection in cystic fibrosis patients [1]. *P*. *aeruginoisa* uses the lung surfactant (which is essential for normal breathing, preventing alveoli collapse and acting as a system of lung defense in the lungs) as a source of nutrients allowing it to colonize a large portion of the lungs. This causes airway plugging and surface damage to epithelial cells [1]. The lung surfactant largely consists of a class of phospholipids called phosphatidylcholine. This phospholipid is a source of fatty acids, which are released during degradation by lipases and phospholipases, which are excreted by *P*. *aeruginosa* [2–4]. These released fatty acids could be broken down through the fatty acid degradation pathway via the β-oxidation cycle [5] to be used as a source of energy or they could be taken up by the cell to be used for other cellular processes.

For instance, upon cellular uptake in other organisms, fatty acids can be metabolized by cytochrome P450 enzymes (CYPs) to produce hydroxy fatty acids, which, in turn, can be used for a variety of physiological functions, including diacid formation [5, 6], sophorolipid production [7], and the synthesis of cutin and suberin in plants [8]. While cytochromes P450 (CYPs) are not directly involved in the β-oxidation cycle of fatty acid degradation, they are essential in forming or initiating the formation of hydroxy fatty acids and/or diacids. These molecules can then act as an energy source as they can be degraded by the β-oxidation cycle [5, 6].

It is unlikely, because of their cellular localization, that fatty acid hydroxylating CYPs in *P*. *aeruginosa* will interact directly with the lung surfactant in order to have access to phosphatidylcholine. Therefore, in order for these CYPs to access the fatty acids released from phosphatidylcholine, *P*. *aeruginosa* expresses all the relevant genes involved in phosphatidylcholine degradation (lipases: LipA and LipC, and phospholipases: PlcH and PlcR). The expression of these genes results in fatty acids, glycerol and phosphorylcholine being released. The individual constituents are further imported and degraded, via high levels of expression of many genes involved in the fatty acid degradation pathway suggesting that *P*. *aeruginosa* may utilize phosphatidylcholine as one of the major nutrient sources *in vivo* [2] and could also be made available for interaction with the CYPs.

In this study, we have investigated CYP168A1 from *P*. *aeruginosa*, the sole member of its cytochrome P450 family, to establish whether this enzyme is able to catalyze the hydroxylation of fatty acids. We have cloned, expressed and purified CYP168A1 and have successfully demonstrated the enzyme’s ability to hydroxylate biologically relevant fatty acids at the sub-terminal carbons.

## Materials and methods

### Chemicals

Growth media, ampicillin, 5-aminolevulenic acid and isopropyl-β-D-thiogalactopyranoside (IPTG) were purchased from Formedium, Ltd. (Hunstanton, UK). Chemicals used in the preparation of phosphate buffers were purchased from Fisher Scientific (Loughborough, UK). Voriconazole was purchased from Discovery Fine Chemicals (Dorset, UK). Palmitoleic acid (C16:1) was purchased from Tokyo Chemical Industry UK Ltd (Oxford, UK). All other fatty acids and chemicals were purchased from Sigma-Aldrich (Poole, UK), unless otherwise stated.

### Heterologous expression and purification of CYP168A1 protein

The *CYP168A1* gene (UniProt accession number Q9I107) was synthesized by Eurofins MWG Operon (Ebersberg, Germany) including nucleotide sequence optimization for expression in *E*. *coli*. The gene was designed to contain the triplet GCT, coding for alanine as the second amino acid, to aid expression in *E*. *coli* and a C-terminus hexahistidine tag to facilitate purification by affinity chromatography using Ni^2+^-NTA agarose. In addition an *Nde*I restriction site was incorporated at the 5’ end and a *Hind*III restriction site at the 3’ end of the gene. The gene was cloned using the *Nde*I and *Hind*III restriction sites into pET17b and transformed into BL21(DE3)pLysS cells under ampicillin and chloramphenicol selection. Transformants were used to inoculate Terrific broth containing ampicillin and grown at 37°C and 180 rpm for 6 hours. 1 mM IPTG and 1 mM 5-aminolevulenic acid were added for induction prior to expression. CYP168A1 was expressed at 25°C and 130 rpm for 20 hours. Cells were harvested (10 min at 3000 x *g*), re-suspended in 0.1 M potassium phosphate buffer (pH 7.4) and stored at -80°C overnight. Samples were thawed and spun at 140000 x *g* for 1 hour at 4°C to recover the solubilized protein in the supernatant. CYP168A1 was purified using Ni^2+^-NTA agarose (Qiagen) and eluted in 0.1 M Tris-HCl (pH 8.1) containing 25% (w/v) glycerol and 1% (w/v) L-histidine. SDS-polyacrylamide gel electrophoresis was undertaken to assess protein purity.

### Determination of cytochrome P450 protein concentration

Reduced carbon monoxide difference spectroscopy was performed (light path, 10 mm) according to the method of Estabrook *et al*., 1972 [9] to determine cytochrome P450 protein concentration using an extinction coefficient of 91 mM^-1^ cm^-1^ at 450 nm [10]. Absolute spectra were determined from 700 nm to 250 nm (light path, 4.5 mm). The heme concentration of the purified CYP168A1, diluted with 10 mM potassium phosphate (pH 7.4), was determined by measuring the Soret peak at 417 nm using an extinction coefficient of 125 mM^-1^ cm^-1^ [11] and the total protein concentration was determined by measurement of the absorbance at 205 nm using an extinction coefficient of 31 ml mg^-1^ cm^-1^ [12] from which the percentage heme incorporation was calculated. The Reinheitszahl (Rz) ratio of absorbance due to the heme Soret peak at 417 nm and that due to the absorbance of the apoprotein was also determined as a primary indicator of enzyme purity and heme incorporation [13]. All spectral determinations were made using a Hitachi U-3310 UV-visible spectrophotometer (San Jose, CA).

### Fatty acid binding studies

Fresh supplies of fatty acids were purchased prior to commencing ligand binding and catalysis studies. Stock solutions containing 0.25 mg ml^-1^ myristic acid (C14:0), palmitic acid (C16:0), stearic acid (C18:0) and oleic acid (C18:1) were prepared in dimethylformamide along with 0.5 mg ml^-1^ palmitoleic acid (C16:1), 0.1 mg ml^-1^ linoleic acid (C18:2) and 10 mg ml^-1^ arachidonic acid (C20:4). These stock fatty acid solutions were progressively titrated against 5 μM of CYP168A1 protein in 0.1 M Tris-HCl (pH 8.1) buffer containing 25% (w/v) glycerol using quartz semi-micro cuvettes with equivalent volumes of dimethylformamide added to the cytochrome P450-containing reference cuvette. Titrations of CYP168A1 with myristic (1.09, 2.19, 3.28, 4.38, 5.47, 6.57, 7.66, 8.076 μM), palmitic (0.98, 1.95, 2.93, 3.9, 4.88, 5.83, 6.83, 7.8 μM), stearic (0.88, 1.76, 2.64, 3.52, 4.39, 5.27, 6.15, 7.03 μM), palmitoleic (0.98, 1.97, 2.95, 3.93, 4.91, 5.90, 6.88, 7.86 μM), oleic (0.89, 1.77, 2.66, 3.54, 4.43, 5.31, 6.20, 7.08 μM), linoleic (0.36, 0.71, 1.07, 1.43, 1.78, 2.14, 2.50, 2.85 μM) and arachidonic (33, 66, 99, 132, 165, 198, 231, 264, 297, 330, 363, 396, 429, 462, 495, 528, 561, 594, 627, 660 μM) acids were performed at room temperature.

The absorbance difference spectra from 500 nm to 350 nm were determined after each incremental addition of fatty acid. Ligand saturation curves were constructed from the change in absorbance (Δ*A*_peak-trough_) against fatty acid concentration. The dissociation constant for the fatty acid-CYP168A1 complex (*K*_s_) was determined by nonlinear regression (Levenberg-Marquardt algorithm) using a rearrangement of the Morrison equation [14] and the Michaelis-Menten equation. The magnitude of the spin state change for type I difference spectra was calculated from Δ*A*_390-420_ using an extinction coefficient of 100 mM^-1^ cm^-1^ [15]. All fatty acid binding experiments were undertaken in quadruplicate.

In between ligand binding determinations, the quartz cuvettes were washed with deionized water and then soaked for 30 min in 2-propanol at room temperature to desorb any residual fatty acids from the cuvette surfaces, followed by rinsing a further three-times with 2-propanol and then deionized water prior to drying.

### Fatty acid reconstitution assays

The reconstitution assay system contained 0.25 μM CYP168A1, 2.5 μM spinach ferredoxin (Sigma-Aldrich F3013), 0.25 μM spinach ferredoxin-NADP^+^ reductase (Sigma-Aldrich F0628), 50 μM dilaurylphosphatidylcholine (DLPC), 100 μM fatty acid, 4 mM glucose-6-phosphate, 3 U/ml yeast glucose-6-phosphate dehydrogenase and 0.1 M potassium phosphate (pH 7.4). Assay mixtures were incubated at 37°C for 5 min prior to initiation with 4 mM β-NADPH-Na_4_ and then incubated for a further 2.5 hours at the same temperature. Fatty acids and their hydroxylated products were recovered by extraction with dichloromethane and dried in a vacuum centrifuge. TMS derivatisation of the samples and analysis by GCMS were performed as previously described [16]. Percentage product formation was calculated from the GC peak areas of the fatty acid and the hydroxylated metabolites, with compound identities confirmed by mass fragmentation patterns of fatty acid standards.

To test the effect of the azole antifungal drugs miconazole, tebuconazole and voriconazole on CYP168A1 turnover, the reconstitution assays were repeated as described above using oleic acid as the substrate, except 2 μM of azole dissolved in DMF was also present. Control assays contained no azole, but an equivalent volume of DMF. Experiments were undertaken in duplicate.

CYP168A1 reconstitution assays were also performed using 50 μM cholesterol, cholesta-4-ene-3-one, progesterone and testosterone as potential substrates. For these assays the CYP168A1 concentration was increased to 2 μM in the presence of 2.5 μM spinach ferredoxin, 0.25 μM spinach ferredoxin-NADP^+^ reductase, 50 μM DLPC, 4 mM glucose-6-phosphate, 3 U/ml yeast glucose-6-phosphate dehydrogenase, 0.1 M potassium phosphate (pH 7.4) and 4 mM β-NADPH-Na_4_, followed by 3 hours incubation at 37°C. Steroid and sterol compounds were extracted with ethyl acetate (2 x 3 ml), dried using a vacuum centrifuge and derivatized firstly with methoxamine followed by silyation using BSTFA-TMCS and analyzed by GCMS(57). Metabolites were identified from GC traces and MS fragmentation patterns compared against positive controls for CYP mediated hydroxylation reactions performed in our laboratory.

### Azole binding studies

The azole antifungal drugs miconazole, tebuconazole and voriconazole were used in binding studies with CYP168A1 in accordance with previously described methods [17, 18]. Stock solutions of these azoles (0.75–10 mg ml^-1^) were prepared in DMF and progressively titrated against 2 μM CYP168A1 in 0.1 M Tris-HCl (pH 8.1) buffer containing 25% (w/v) glycerol. Equivalent volumes of DMF were added to a reference cuvette containing 2 μM of CYP168A1. Titrations with miconazole (1.80, 3.60, 5.41, 7.21, 9.01, 10.81, 12.61 μM), tebuconazole (16.24, 32.49, 48.73, 64.97, 81.22, 97.46, 113.70 μM) and voriconazole (28.63, 57.26, 85.88, 114.51, 143.14, 171.77, 200.40, 229.02, 257.65 μM) were performed at room temperature.

The difference spectrum from 500 nm to 350 nm was determined after each incremental addition of azole. Binding saturation curves were constructed from the Δ*A*_peak-trough_ against azole concentration. The dissociation constant (*K*_d_) of the enzyme-azole complex was determined by nonlinear regression (Levenberg-Marquardt algorithm) using a rearrangement of the Morrison equation [14]. All binding experiments were undertaken in duplicate.

### MIC determinations

*P*. *aeruginosa* strains DSMZ 22644 and ATCC 39324 were grown in LB media overnight. Optical density at 520 nm of the overnight cultures was measured using a spectrophotometer. Cultures were adjusted to give an optical density of approximately 0.2, which is equivalent to a cell count of approximately 1 x 10^9^. The cells were pelleted and resuspended in the same volume of water. Further dilutions were made in M9 media with oleic acid used as the carbon source (instead of glucose) to give approximately 2 x 10^5^ cells. Stock concentrations of tebuconazole, miconazole and voriconazole were prepared in dimethyl sulfoxide (12.8, 6.4, 3.2, 1.6, 0.8, 0.4, 0.2, 0.1, 0.05 and 0.025 mg ml^-1^). These stock azole solutions were diluted ten-fold in fresh LB media and then diluted a further ten-fold with inoculum in microtiter plate wells. This gave final azole concentrations of 128, 64, 32, 16, 8, 4, 2, 1, 0.5 and 0.25 μg ml^-1^. The microtiter plates were incubated at 37°C for 24 hours. Following this initial incubation period, 20 μl of 0.2% Resazurin was added to each microtiter plate well. The plates were incubated for a further 48 hours at 37°C before being read. A color change from purple to pink indicated the presence of respiring cells. Each azole MIC determinations were performed in triplicate and scored manually.

### Data analysis

Curve-fitting of ligand binding data were performed using the computer program QuantumSoft ProFit (version 6.1.12). Phylogenetic analysis of CYP168A1 was performed using the UniProt BLAST online software resource (http://www.uniprot.org/blast/). Amino acid sequence alignments were performed using ClustalX version 1.81 software (http://www.clustal.org/).

## Results

### Heterologous expression of CYP168A1

**Fig 1:**
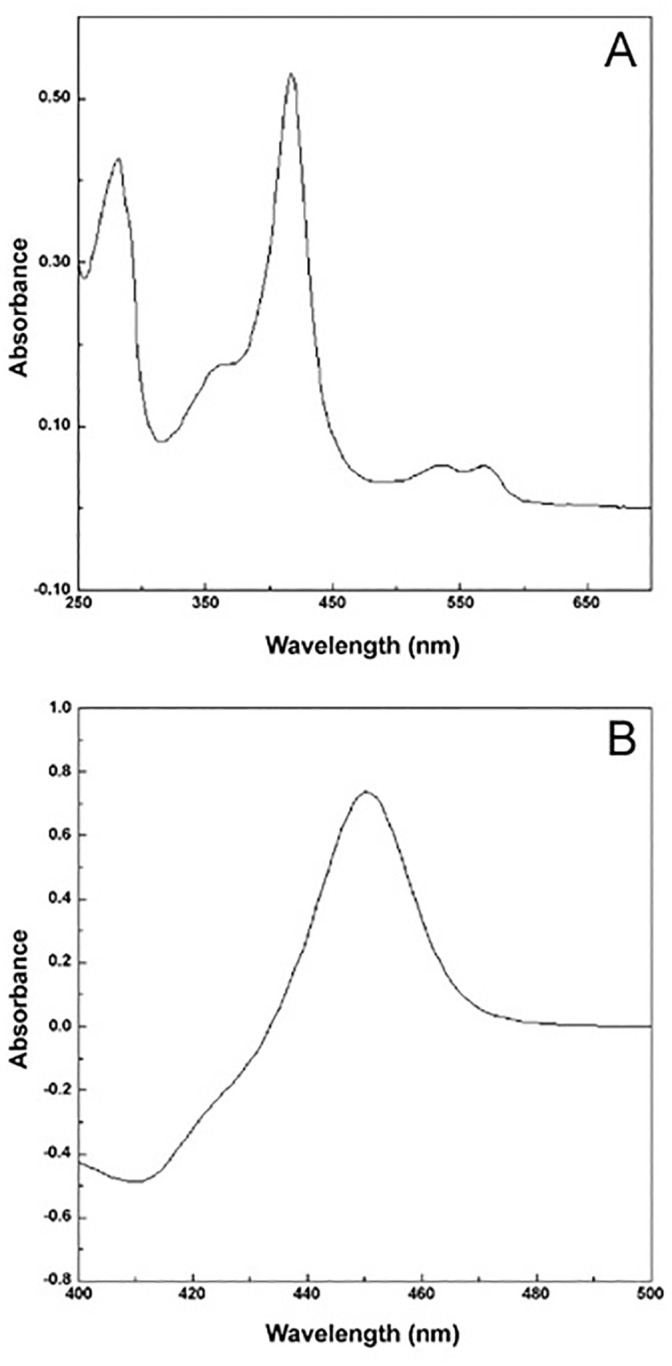
Spectral properties of CYP168A1. The absolute spectrum of a twenty-fold dilution of purified CYP168A1 in the oxidized resting state (4.5 mm light path) is shown (A) in addition to the dithionite-reduced carbon monoxide difference spectrum (light path 10 mm) of 9 μM purified CYP168A1 (B).

Overexpression of CYP168A1 in *E*. *coli* resulted in protein yields of ∼900 nmol per liter of culture. SDS-polyacrylamide gel electrophoresis, following Ni^2+^-NTA agarose purification, confirmed that CYP168A1 was over 90% pure as judged by staining intensity with Coomassie brilliant blue R-250. The absolute spectrum of CYP168A1 (Fig 1A) was characteristic of a ferric cytochrome P450 enzyme that had been isolated predominately in the low-spin state with a Soret peak at 417 nm [19, 20]. The A_393-470_/A_417-470_ value was 0.412, where 0.4 is indicative of 100% low-spin occupancy and 2.0 indicative of 100% high-spin occupancy [21], confirming CYP168A1 to be over 95% low-spin in the oxidized resting state. The observed A_417_/A_280_ for CYP168A1 was 1.26, which was within the expected range of 1 and 2 for a cytochrome P450 [13]. Heme incorporation was 77 to 81% calculated from measurements at A_205_ and A_417_ [11, 12] with a specific heme content of 15.3 nmol mg^-1^ protein comparable to the expected value of 17 to 20 nmol mg^-1^ for cytochrome P450 enzymes [13]. The dithionite-reduced carbon monoxide difference spectrum for CYP168A1 (Fig 1B) was characteristic of CYPs isolated in their native state exhibiting a red-shifted Soret peak at 450 nm [9, 10].

### Phylogenetic analysis of CYP168A1

*P*. *aeruginosa* strain PAO1 contains three putative cytochrome P450 monooxygenases PA2475, PA3331 and PA3679 (https://www.pseudomonas.com/) and these have been assigned as CYP168A1, CYP107S1 and CYP169A1 (https://drnelson.uthsc.edu/) with corresponding UniProt accession numbers Q9I107, Q9HYR4 and Q9HXW1, respectively.

Phylogenetic analysis using BLASTP indicated CYP168A1 to be confined to *Acinetobacter baumannii*, *Enterobacter cloacae* and *P*. *aeruginosa* (sequence identities 98.2 to 99.8%) with some genes putatively described as ’biotin biosynthesis cytochrome P450 bioI’. The latter ’bioI’ genes included *A*. *baumannii* UniProt accession number A0A1G5LRV6 (99.8% sequence identity) and *P*. *aeruginosa* A0A0P1DBG8 and A0A0F7QM08 (both 99.8% sequence identities). Sequence identity falls to 35–42% for the next closest group of cytochrome P450 enzymes, which feature numerous CYPs from *Streptomyces sp*., and CYPs from *Candidatus Rokubacteria bacterium*, *Allokutzneria albata*, *Microcystis aeruginosa*, *Scytonema hofmannii*, *Amycolatopsis orientalis*, *Saccharomonospora cyanea*, *Nitrolancea hollandica*, *Actinomadura madurae*, *Kutzneria albida*, and *Actinobacteria bacterium*. Nearly all of these CYPs were of unknown function, but some were assigned putative functions of Linalool 8-monooxygenase, CYP107B1, and peroxidase enzymes. Alignment of the CYP168A1 amino acid sequence against CYP107H1 enzymes from *Bacillus subtilis* (P53554), *Bacillus amyloliquefaciens* (Q70JZ2) and *Bacillus licheniformis* (Q65MK8) showed that only 9 out of the 32 residues associated with CYP107H1 function in *Bacillus sp*. [22] were also conserved in CYP168A1 (excluding the heme-coordinating cysteine residue) and that CYP168A1 shared only 31% sequence identity with the *Bacillus* CYP107H1 enzymes. Therefore, CYP168A1 did not appear to be a CYP107H homolog.

### Fatty acids are able to bind to CYP168A1

The heme prosthetic group of CYPs exists in equilibrium between the low-spin (hexacoordinate) form, characterized by a heme Soret peak at ∼418 nm, and the high-spin (pentacoordinate) form, characterized by a heme Soret peak at ∼393 nm. The resting state of most CYPs is predominantly the low-spin ferric form [23]. Ligand binding can perturb the spin state equilibrium in two main ways. Substrates and other ligands can bind to the CYP (though the ligands themselves do not directly coordinate to the heme) resulting in the displacement of the axial-ligated water molecule from the heme ferric ion and a change in spin state from low- to high-spin. The magnitude of the spin state change observed is dependent on both the CYP and the ligand. For example, lauric acid binding to *Streptomyces peucetius* CYP147F1 causes 95% of the enzyme molecules to undergo a low- to high-spin state transition [24], whereas for eukaryotic CYP51 enzymes the spin state changes associated with substrate binding rarely exceed 10% [25]. This low- to high-spin transition gives rise to a type I difference spectrum that is characterized by a spectral peak at ∼390 nm and trough at ∼420 nm. Other ligands can directly coordinate to the heme ferric ion (commonly through an aromatic nitrogen atom) [19, 26], for example the binding of azole antifungals to CYP51 enzymes [27]. The resultant complexes are low-spin (hexacoordinate) and often result in a red-shift of the Soret peak from 418 nm to 425–434 nm [19]. This direct coordination of the ligand to the heme ferric ion gives rise to a type II difference spectrum [19]. The spectral peak varies from 425 nm (if the CYP was 100% in the high-spin state before ligand binding) to 432 nm (if the CYP was 100% in the low-spin state) with respective troughs of 390 nm and 410 nm. For CYPs of mixed spin state the peaks and troughs will be at intermediate wavelengths.

**Fig 2:**
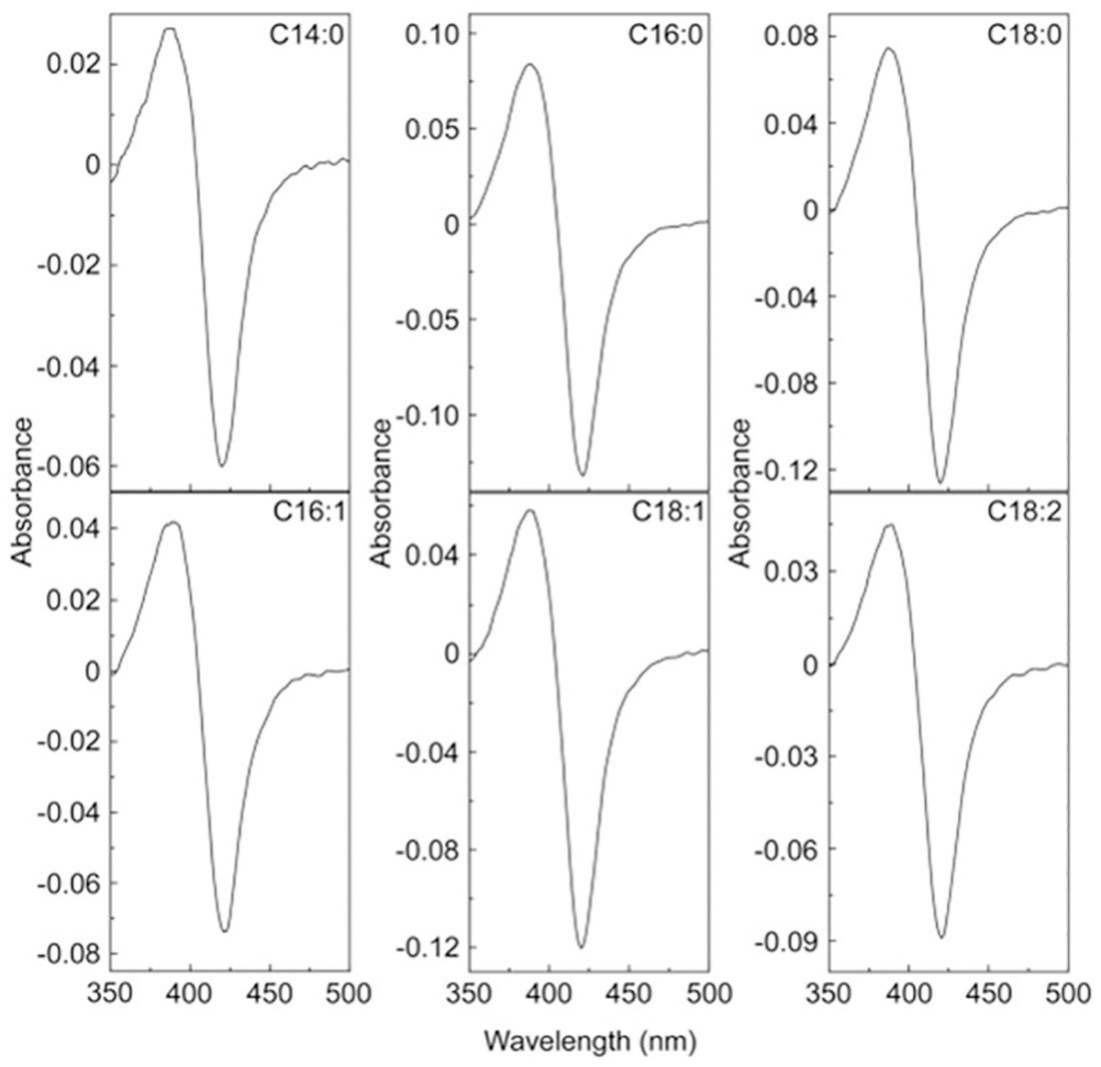
Type i absorbance difference spectra. Absorbance difference spectra were determined using 0.5 μg ml^-1^ myristic (C14:0), palmitic (C16:0), stearic (C18:0), palmitoleic (C16:1), oleic (C18:1) and linoleic (C18:2) acids and 5 μM CYP168A1 in quartz semi-micro cuvettes of 10 mm path length at room temperature. Composite type I difference spectra and saturation curves for the titration of each fatty acid can be found in Fig 3.

**Fig 3:**
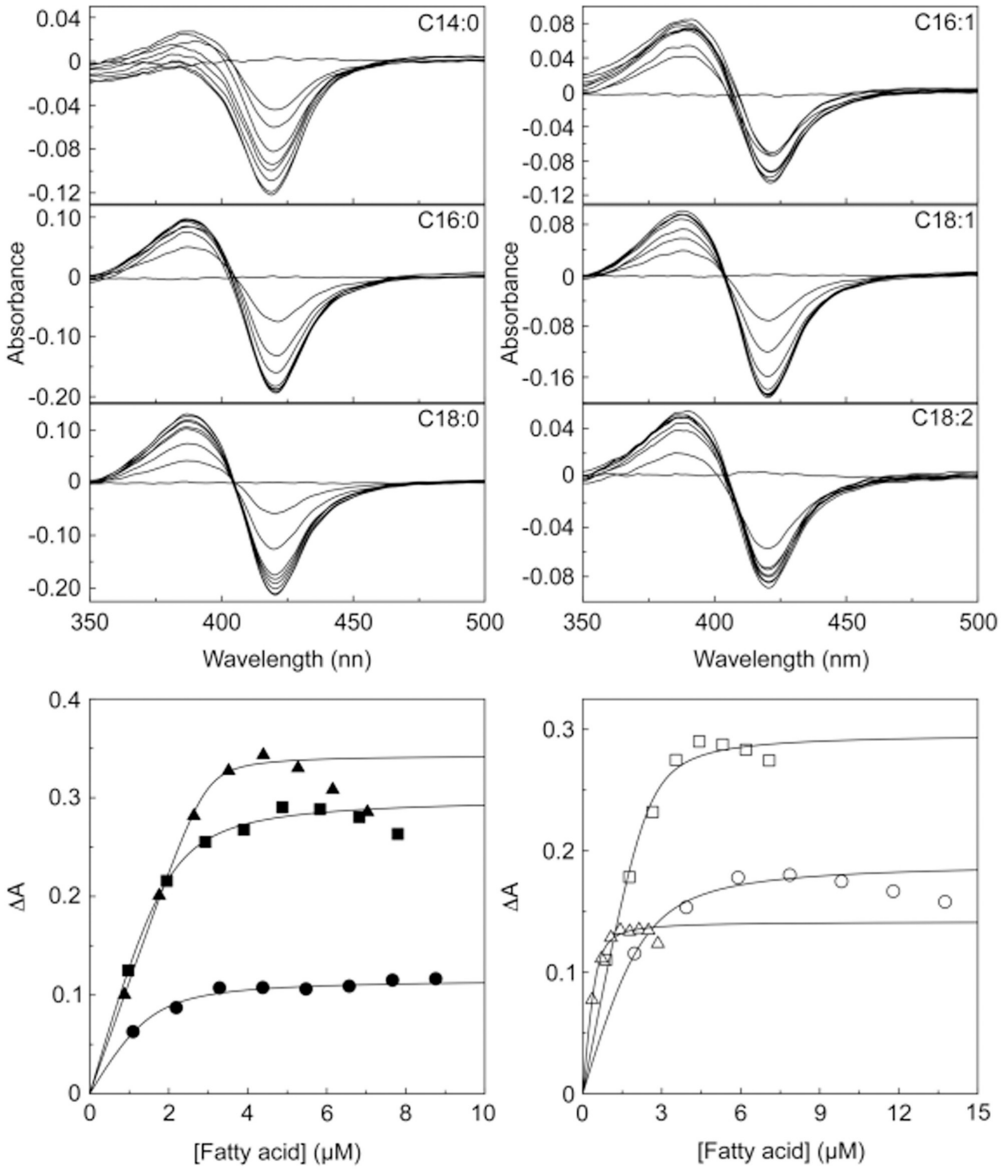
Fatty acid binding to CYP168A1. Myristic, palmitic, stearic and oleic acids (0.25 mg ml^-1^ in DMF), palmitoleic acid (0.5 mg ml^-1^ in DMF) and linoleic acid (0.1 mg ml^-1^ in DMF) were progressively titrated against 5 μM CYP168A1 in quartz semi-micro cuvettes of path length 10 mm. After each 1 μl addition of fatty acid the difference spectrum was measured against a CYP168A1-containing reference cuvette in which an equivalent volume of DMF was added. Ligand saturation curves for myristic (filled circles), palmitic (filled squares), stearic (filled triangles), palmitoleic (empty circles), oleic (empty squares) and linoleic (empty triangles) acids were constructed and fitted using a rearrangement of the Morrison equation [14]. Ligand binding experiments were performed in quadruplicate, although only one replicate is shown.

**Fig 4:**
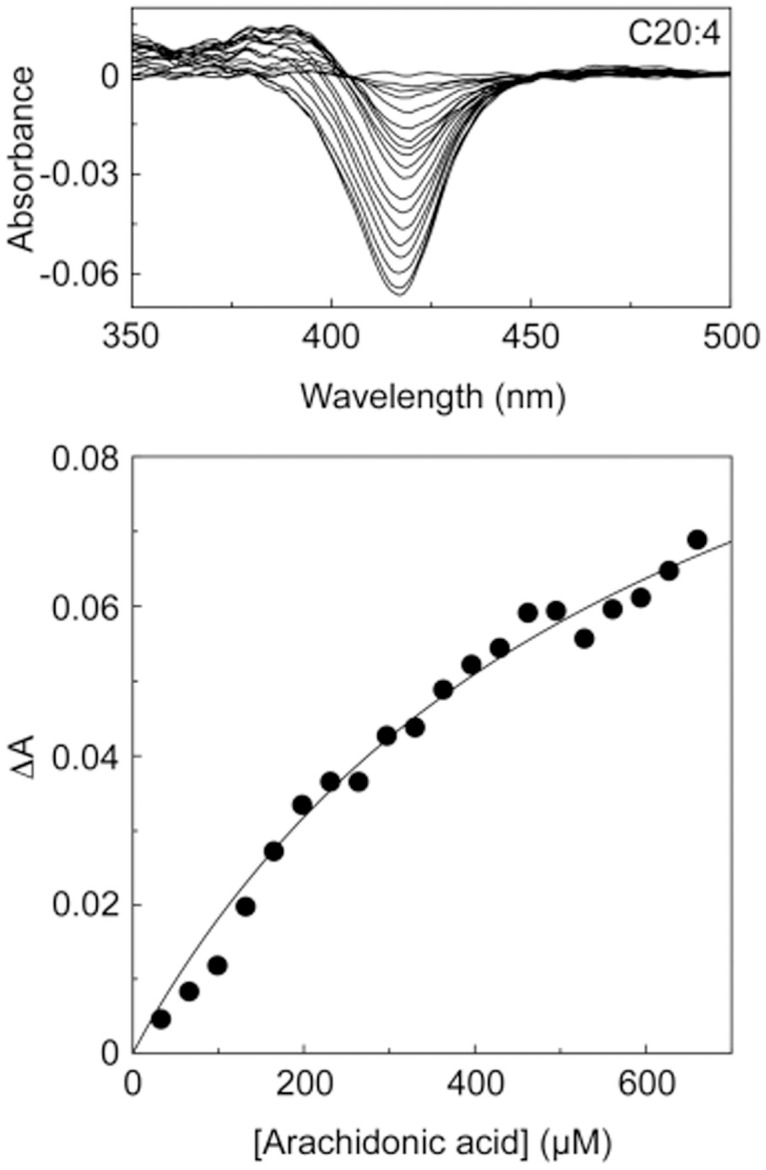
Arachidonic acid binding to CYP168A1. Arachidonic acid (10 mg ml^-1^ in DMF) was progressively titrated against 5 μM CYP168A1 in quartz semi-micro cuvettes of path length 4.5 mm. After each 1 μl addition of arachidonic acid the difference spectrum was measured against a CYP168A1-containing reference cuvette in which an equivalent volume of DMF was added. The ligand saturation curve was constructed and fitted using the Michaelis-Menten equation.

Type I absorption difference spectra were obtained for all the fatty acids with 5 μM CYP168A1. The peak at 393 nm and trough at 418 nm is most clearly shown for the difference spectrum obtained with 0.5 μg ml^-1^ fatty acid and 5 μM CYP168A1 (Fig 2), equivalent to 2.19, 1.95, 1.76, 1.97, 1.77 and 1.78 μM for myristic, palmitic, stearic, palmitoleic, oleic and linoleic acid, respectfully. The cumulative difference spectra obtained during the ligand titration for myristic acid (C14:0), palmitic acid (C16:0), palmitoleic acid (C16:1), stearic acid (C18:0), oleic acid (C18:1), and linoleic acid (C18:2) and the associated ligand saturation curves are shown in Figs 3 and 4 for arachidonic acid (C20:4). These type I difference spectra suggest that all seven fatty acids were potential substrates for CYP168A1. Spectral isosbestic points were observed for palmitic (404 nm), stearic (404 nm), palmitoleic (406 nm), oleic (403 nm) and linoleic (406 nm) acids. However, no clear isosbestic points were observed for myristic and arachidonic acids due to the spectral peak at ∼393 nm progressively diminishing above a threshold fatty acid concentration whilst the spectral trough at ∼421 nm continued to deepen.

It was important to optimize the stock fatty acid concentrations so as to obtain sufficient ascendant points on the titration curve before reaching ligand saturation to facilitate curve fitting of data and accurate *K*_s_ determination, especially as the type I difference spectra for some fatty acids (notably stearic, palmitoleic and oleic acids) started to dissipate at higher ligand concentrations (Fig 3). This may be caused by either slow aggregation of the CYP168A1-fatty acid complex (although no visible precipitation was observed) or a perturbation of the spin-state equilibrium of the CYP168A1-substrate complex so that some molecules transition to the low-spin state by partial coordination of a water molecule as the sixth axial heme ligand. This phenomenon was evident in the ligand saturation curve for palmitic acid with CYP168A1 reported by Tooker et al [28].

This phenomenon of partial dissipation of type I binding spectra at higher ligand concentrations had previously been observed for cholesterol (but not 4-cholesten-3-one) binding to CYP125 from *Mycobacterium tuberculosis* [29]. Capyk et al [29] demonstrated this phenomenon was due to the ligand solvent (10% w/v 2-hydroxypropyl-β-cyclodextrin), as when the solvent was changed (to 25 mM EDTA-bridged β-cyclodextrin dimer) the premature dissipation of the type I difference spectrum was no longer observed with cholesterol. As the partial dissipation of type I binding spectra appears to be both ligand- and solvent-specific, both molecules must interact with the CYP protein to cause this effect. When the fatty acid solvent was changed from DMF to ethanol the partial dissipation of the type I binding spectra still occurred at higher ligand concentrations.

The binding saturation curves for all the fatty acids, except arachidonic acid, were best fit using a rearrangement of the Morrison equation [14] (Table 1, Fig 3) to calculate the dissociation constant of the substrate-CYP168A1 complex (*K*_s_), indicating tight binding to CYP168A1. The Michaelis-Menten equation gave the best fit for arachidonic acid binding (Fig 4). Stearic and linoleic acids bound the tightest to CYP168A1 with apparent *K*_s_ values under 0.05 μM, followed by palmitic acid (*K*_s_ 0.12 μM), then oleic and palmitoleic acids (*K*_s_ ∼0.15 μM) and myristic acid (*K*_s_ 0.25 μM), with arachidonic acid binding having the lowest affinity (*K*_s_ ∼460 μM). The fold-difference in apparent *K*_s_ values compared to stearic and linoleic acids were ∼2-, ∼3-, ∼3- ∼5-, and ∼9160-fold for palmitic, oleic, palmitoleic, myristic, and arachidonic acids, respectively. The high *K*_s_ value for arachidonic acid suggested it would be a poor CYP168A1 substrate. Therefore, based on apparent ligand binding affinities, CYP168A1 exhibits a preference for C16 to C18 fatty acids. The magnitude of the low- to high-spin transitions induced were 23, 58, 37, 66, 55, 28 and 32% for myristic, palmitic, palmitoleic, stearic, oleic, linoleic and arachidonic acids, respectively, based on the observed ΔA_max_ values (Table 1).

**Table 1:** Fatty acid binding affinities for CYP168A1.

Fatty acid	*K*_s_ (μM)	ΔA_max_	Low- to high-spin state change (%)
Myristic acid	0.249 ±0.103	0.144 ±0.08	22.8 ±1.6
Palmitic acid	0.117 ±0.056	0.290 ±0.015	58.1 ±3.0
Palmitoleic acid	0.155 ±0.083	0.185 ±0.006	37.0 ±1.2
Stearic acid	<0.05 ^a^	0.328 ±0.020	65.6 ±4.1
Oleic acid	0.142 ±0.012	0.277 ±0.014	55.4 ±2.8
Linoleic acid	<0.05 ^a^	0.142 ±0.013	28.3 ±2.6
Arachidonic acid	458 ±87	0.071 ±0.009 ^b^	31.6 ±4.0 ^b^

Type I difference spectra were observed for all seven fatty acids with 5 μM CYP168A1.

Mean *K*_s_ values from four replicates were calculated using a rearrangement of the Morrison equation [14] and are shown ± standard deviations, except for arachidonic acid where the Michaelis-Menten equation was used.

^a^ The *K*_s_ values for these two fatty acids were below the lower accuracy limit of the Morrison equation of 0.05 μM (1% the concentration of the enzyme) [61].

^b^ Path length of cuvettes used with arachidonic acid were 4.5 mm compared to 10 mm used with the other fatty acids.

Binding saturation was not achieved at 660 μM arachidonic acid.

### CYP168A1 preferentially hydroxylates fatty acids at the ω-1 carbon

**Fig 5:**
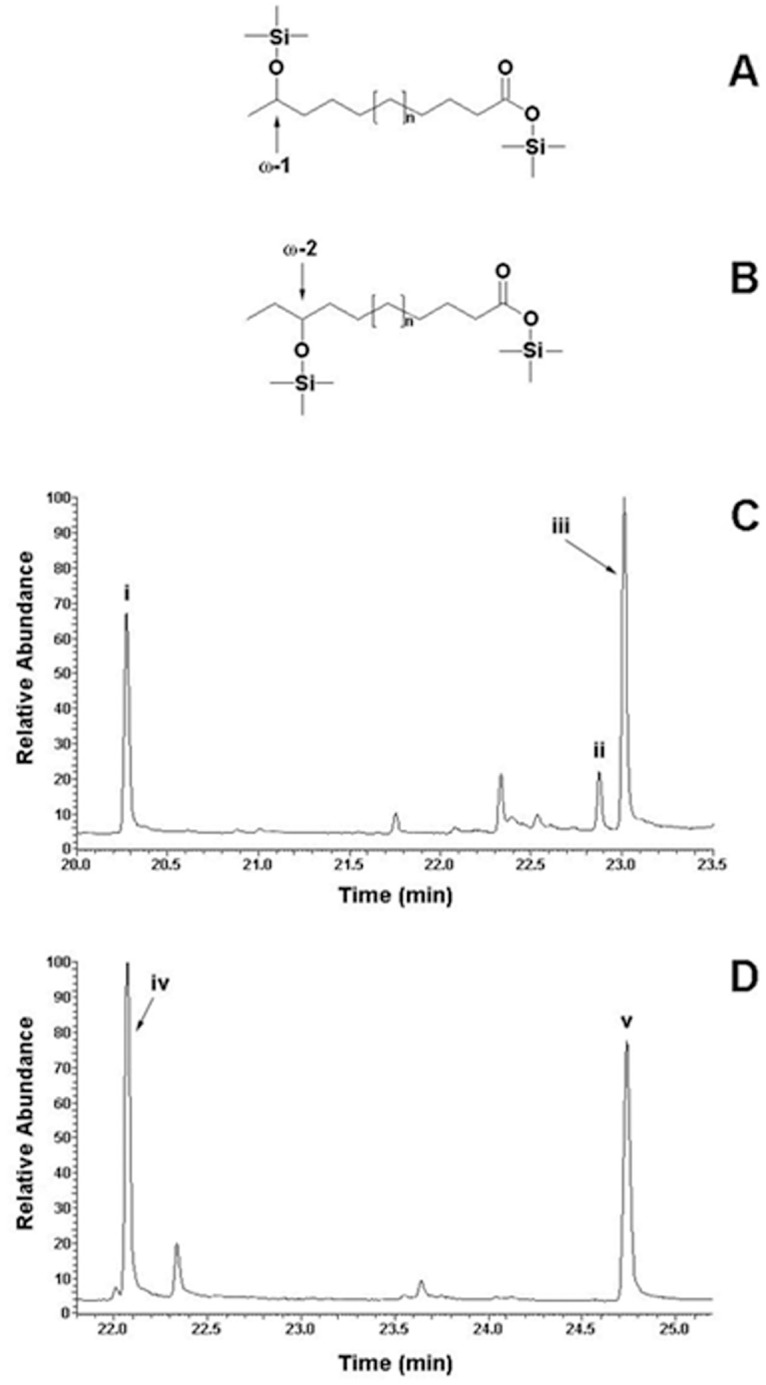
Gas chromatograms of CYP168A1 assay metabolites. The chemical structures of TMS-derivatised ω-1-hydroxy fatty acids (A) and ω-2-hydroxy fatty acids (B) are shown. Gas chromatograms for TMS-derivatized assay metabolites obtained when palmitic acid (C) and oleic acid (D) were used as substrates are shown. Peak (i) corresponds to palmitic acid, peak (ii) to ω-2-hydroxypalmitic acid, peak (iii) to ω-1-hydroxypalmitic acid, peak (iv) to oleic acid and peak (v) to ω-1-hydroxyoleic acid (all TMS-derivatives). Mass fragmentation patterns of the palmitic acid products can be found in the supporting information (S1 Fig). Other minor GC peaks were identified as impurities present in the source fatty acids.

CYP168A1 was able to catalyze the hydroxylation of the saturated fatty acids myristic acid, palmitic acid, and stearic acid at both the ω-1- and ω-2-carbons (Fig 5A and 5B), showing preference for the ω-1-carbon (Table 2). In this assay system, CYP168A1 gave the highest catalytic turnover with palmitic acid resulting in the formation of ω-1 hydroxypalmitic acid at 1.27 min^-1^ and ω-2 hydroxypalmitic acid at 0.34 min^-1^ (Fig 5C; Table 2). Using palmitic acid as substrate resulted in 4.7- and 1.2-fold more ω-1-hydroxy product and 10.3- and 2-fold more ω-2-hydroxy product being formed than when myristic acid and stearic acid were used, respectively (Table 2). However, the proportion of myristic acid converted to ω-1-hydroxy product than ω-2-hydroxy product was substantially higher than observed for the other two saturated fatty acids. The mass fragmentation patterns of the CYP168A1 metabolites ω-1-hydroxypalmitic, ω-2-hydroxypalmitic and ω-1-hydroxyoleic acids are shown in S1 Fig. The mass fragmentation patterns for the TMS-derivatised fatty acid standards can be found in the publication by Williams et al. [30].

**Table 2:** Fatty acid hydroxylation by CYP168A1.

Fatty acid	Carbon hydroxylated	% Product ^a^	Turnover no. (min^-1^)
Myristic acid	ω-1-	11 ± 0.6	0.27 ± 0.02
	ω-2-	1.3 ± 0.03	0.033 ± 0.001
Palmitic acid	ω-1-	49 ± 3.8	1.27 ± 0.10
	ω-2-	13 ± 4.0	0.34 ± 0.10
Palmitoleic acid	ω-1-	15 ± 2.4	0.38 ± 0.06
	ω-2-	-	-
Stearic acid	ω-1-	41 ± 2.9	1.07 ± 0.09
	ω-2-	6.6 ± 0.5	0.17 ± 0.01
Oleic acid	ω-1-	49 ± 1.2	1.28 ± 0.03
	ω-2-	-	-
Linoleic acid	ω-1-	14 ± 1.3	0.35 ± 0.03
	ω-2-	-	-

Mean values from two replicates ± standard error are presented.

^a^ percentage of substrate converted into hydroxylated product after 2.5 h incubation at 37°C. The starting substrate and CYP168A1 concentrations were 100 μM and 0.25 μM, respectively. No hydroxylated products were obtained when 100 μM arachidonic acid was used as substrate.

CYP168A1 was also able to hydroxylate the unsaturated fatty acids palmitoleic acid, oleic acid and linoleic acid, but only at the ω-1-carbon. Catalytic turnover with oleic acid was similar to that of palmitic acid in terms of production of the ω-1-hydroxy metabolite ω-1-hydroxyoleic acid (1.28 min^-1^) (Fig 5D, Table 2) and was 3.4- and 3.7-fold greater than the relative ω-1-hydroxy metabolites produced when palmitoleic and linoleic acids were used as substrates. Lower catalytic turnovers were observed with palmitoleic and linoleic acids (0.38 and 0.35 min^-1^, respectively) although both these fatty acids bound tightly to CYP168A1 (*K*_s_ values of 0.155 μM and >0.05 μM, respectively). Reconstitution assays with arachidonic acid showed no detectable product formation and a large *K*_s_ value of 458 μM was obtained for arachidonic acid with CYP168A1 (Table 1). No C-C cleavage of fatty acyl chains was detected in the assay products, suggesting that CYP168A1 was not a CYP107H homolog.

Catalytic turnover established the order of substrate preference to be palmitic acid (highest turnover) followed by oleic acid, stearic acid, palmitoleic acid, linoleic acid and finally myristic acid with the lowest turnover (5.3-fold lower than palmitic), whilst arachidonic acid was catalytically inactive. In contrast, the fatty acid *K*_s_ data suggested the order of substrate preference would have been stearic and linoleic acids, with the lowest *K*_s_ values, followed by oleic acid, palmitoleic acid, myristic acid, and finally arachidonic acid with a 9000-fold larger *K*_s_ value than stearic acid. Both stearic and linoleic acids gave similar apparent *K*_s_ values with CYP168A1 and yet the catalytic turnover observed with stearic acid was 3.5-fold greater than that for linoleic acid. Further investigations are required in order to determine the mechanisms responsible for the observed differences in CYP168A1 catalytic turnover between substrates.

CYP168A1 reconstitution assays using cholesterol, cholesta-4-ene-3-one, progesterone and testosterone as potential substrates gave no oxygenated products, suggesting CYP168A1 is a fatty acyl hydroxylase.

### Azoles are able to bind to CYP168A1, but they have no effect on *P*. *aeruginosa* growth

**Fig 6:**
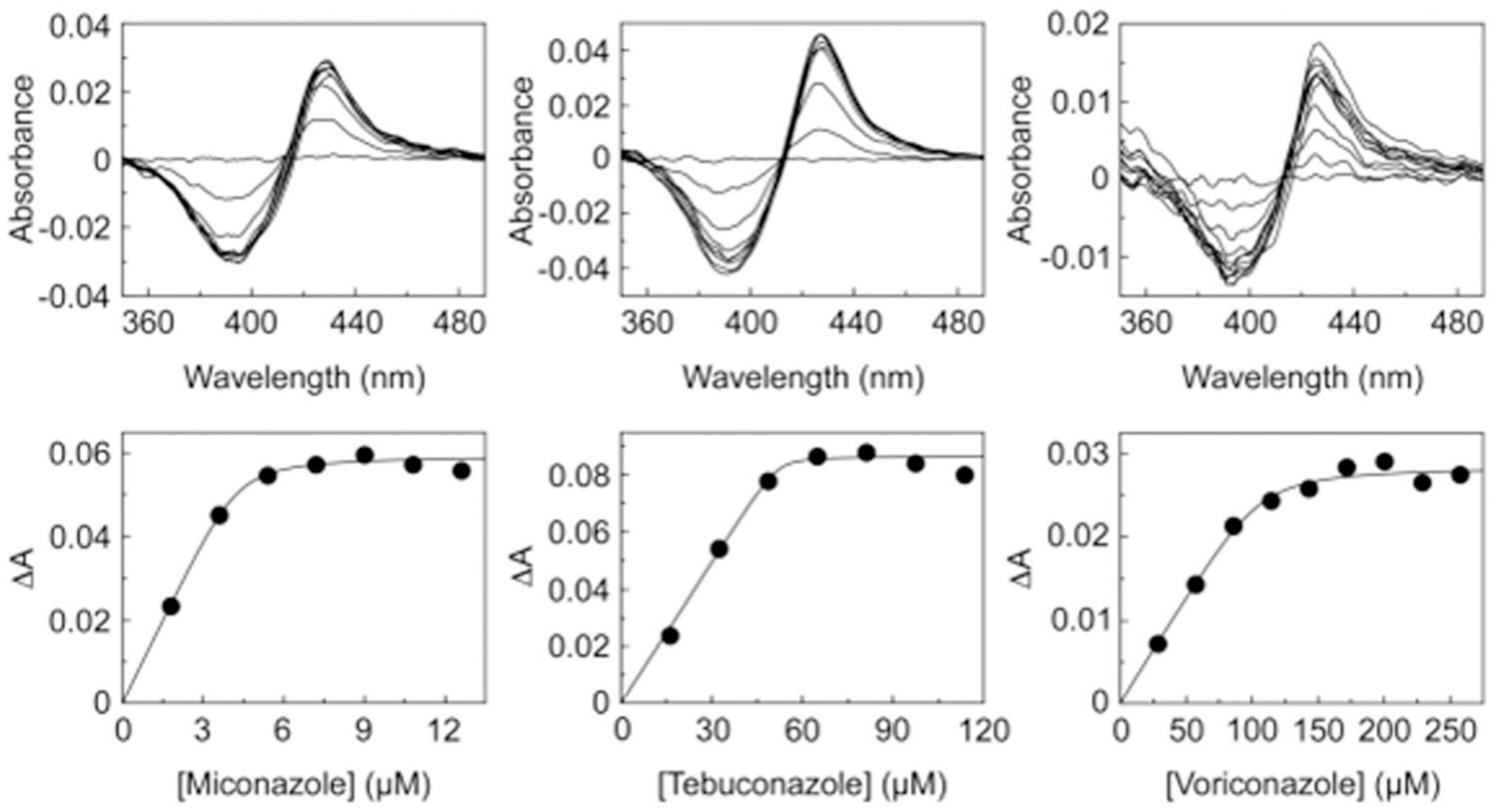
Binding of azole antifungals to CYP168A1. Miconazole (0.75 mg ml^-1^), tebuconazole (5 mg ml^-1^) and voriconazole (10 mg ml^-1^) were progressively titrated against 2 μM CYP168A1 in quartz semi-micro cuvettes of path length 4.5 mm. After each 1 μl addition of azole solution the difference spectrum was measured against a CYP168A1-containing reference cuvette in which an equivalent volume of DMF was added. The cumulative type II difference spectra are shown along with the ligand saturation curves which were fitted using a rearrangement of the Morrison equation [14].

CYP168A1 was titrated against the azole antifungal drugs tebuconazole, voriconazole and miconazole. The enzyme was able to bind each of the three azoles, eliciting type II difference spectra (Fig 6), with apparent dissociation constants (*K*_d_) for the azole-CYP168A1 complexes of 0.18 ±0.01, 2.05 ±0.54 and 0.19 ±0.06 μM for tebuconazole, voriconazole and miconazole, respectively, calculated from the ligand saturation curves (Fig 6). This was in contrast to *K*_d_ values for tebuconazole, voriconazole and miconazole obtained with *Candida albicans* CYP51 of 0.036, 0.010 and 0.026 μM, respectively [27, 31]. The addition of 2 μM azoles to reconstitution assays containing 0.25 μM CYP168A1 showed they elicited no effect on the catalytic activity towards oleic acid when compared against the DMF control. However, the presence of 0.5% DMF in the CYP168A1 assay system caused a 10-fold reduction in oleic acid turnover.

Tebuconazole, voriconazole and miconazole were used in minimum inhibitory concentration (MIC) determinations with *P*. *aeruginosa* strains DMZ 22644 and ATCC 39324 grown on oleic acid. In all cases, the azoles at concentrations up to 128 μg ml^-1^ had no inhibitory effect on *P*. *aeruginosa* growth as the Resazurin indicator turned pink in all wells, indicating cell respiration.

## Discussion

Fatty acids are essential for cell life. Hydroxylation of these molecules can result in the formation of hydroxy fatty acids that can act as signaling molecules and can be used to produce more complex molecules, such as diacids [7, 32]. Therefore, identification and characterization of enzymes involved in fatty acid metabolism is important, particularly in pathogens, such as *P*. *aeruginosa*, where insight into their function can be used to identify new potential targets for novel inhibitors and teach us more about the mechanism of fatty acid degradation.

CYP168A1 was able to bind a range of biologically relevant fatty acids. The *K*_s_ range of <0.05 to 0.25 μM for fatty acids that were catalytically active with CYP168A1 were similar to those observed previously for *Streptomyces peucetius* CYP147F1 [24], *Sorangium cellulosum* CYP267A1 [33], *Streptomyces coelicolor* A3(2) CYP105D5 [34], and some studies with *Bacillus megaterium* CYP102A1 [35]. The *K*_s_ values in this study were similar to those recently reported for *P*. *aeruginosa* CYP168A1 [28], with the exception for stearic acid which was over 6-fold lower (<0.05 μM compared to 0.327 μM) and arachidonic acid which was over 400-fold higher (458 μM compared to 0.96 μM) with the latter mainly due to the atypical difference spectrum observed with arachidonic acid in this study. Higher *K*_s_ values have been observed (8 to 30 μM) with *Bacillus subtilis* CYP107H1 [36] and substantially higher *K*_s_ values (19 to 1065 μM) observed with *Bacillus subtilis* CYP102A2 and CYP102A3 [37].

CYP168A1 fatty acid turnover rates were similar to those obtained with *Sphingomonas paucimobilis* CYP152B1 [38], with both CYPs giving highest turnovers with palmitic acid, and with *Sorangium cellulosum* CYP267A1 [33], except the highest turnover was obtained for capric acid with CYP267A1. Fatty acid turnover rates with CYP168A1 were ∼10-fold higher than those observed with *Mycobacterium marinum* CYP153A16 and *Marinobacter aquaeolei* CYP153A [39] and turnover of palmitic acid was 23-fold greater than observed with *Mycobacterium tuberculosis* CYP124 [40]. The CYP168A1 turnover numbers obtained in this study, although being relatively low for cytochrome P450 monooxygenases, were similar to those reported by Tooker et al [28] of 0.138 min^-1^ with lauric acid and 0.222 min^-1^ with arachidonic acid, however, in this study no catalytic activity was observed with arachidonic acid. At present we cannot account for this difference, although solubility / bioavailability of arachidonic acid in the in vitro reconstitution assay may be a contributory factor. Interestingly, Tooker et al [28] CYP168A1 *K*_m_ for lauric acid was 25-fold higher than the *K*_s_ value, suggesting that other parameters besides substrate binding affinity were significant contributors to the observed *K*_m_ value.

In contrast, CYP168A1 fatty acid turnovers were 10- to 30-fold lower than observed with *Streptomyces peucetius* CYP147F1 [24] and *Candida albicans* CYP52A21 [41], and 25- to 10000-fold lower than observed with *Bacillus subtilis* CYP102A2 and CYP102A3 [37], *Fusarium oxysporum* CYP505 [42], and *Bacillus megaterium* CYP102A1 [35]. *S*. *coelicolor* CYP105D5 fatty acid turnover was greatest with lauric, oleic and arachidonic acids, but gave low turnovers with myristic and palmitic acids [34]. In contrast CYP168A1 exhibited greatest turnover with palmitic, stearic and oleic acids. Therefore, CYP168A1 fatty acid turnover rates were similar to some bacterial CYP fatty acid hydroxylases but are substantially lower than others, especially the CYP102 family, and are 50- to 100-fold lower than the rates of lauric acid hydroxylation observed with human CYP4A1 and CYP4A3 [43, 44].

CYP168A1 from *P*. *aeruginosa* is able to hydroxylate biologically relevant saturated fatty acids at both the ω-1- and ω-2-positions, while it can only hydroxylate unsaturated fatty acids at the ω-1-position. Fatty acid terminal and sub-terminal hydroxylating CYPs from other bacteria tend to be able to hydroxylate fatty acids at a wider range of positions. For instance, CYP107H1 from *Bacillus subtilis* has been shown to hydroxylate myristic acid at the ω-1-, ω-2- and ω-3-carbons, and at the ω-1-, ω-2-, ω-3-, ω-4- and ω-5-carbons in palmitic acid [45]. Interestingly, CYP107H1 preferentially hydroxylates myristic acid at the ω-3-carbon and showed similar hydroxylation at all 5 positions noted in palmitic acid [45], whereas CYP168A1 was shown to preferentially hydroxylate at the ω-1-postion regardless of chain length. The inability of CYP168A1 to metabolize cholesterol, cholesta-4-ene-3-one, progesterone and testosterone suggests the enzyme is a fatty acyl hydroxylase.

Not all bacterial fatty acid hydroxylating CYPs only hydroxylate unsaturated fatty acids at one position. For instance, CYP105D5 can hydroxylate oleic acid at multiple positions with a preference for the ω-1-position [34]. CYP102A1 can also cause the epoxidation of unsaturated fatty acids, but no epoxidation was observed when CYP168A1 was used in reconstitution assays with unsaturated fatty acids, such as oleic acid and linoleic acid. In the presence of arachidonic acid, CYP102A1 is able to both hydroxylate at the ω-2-carbon and cause epoxidation between carbons 14 and 15 [46], whereas CYP168A1 showed no turnover of arachidonic acid in this study.

In all cases, CYP168A1 was only able to hydroxylate the fatty acids at the sub-terminal carbons, with no activity observed at the terminal, ω- carbon, unlike other bacterial fatty acid hydroxylating CYPs, such as CYP124 (36) and CYP119 from *Sulfolobus acidocaldarius* [47], and those from eukaryotes, such as members of the CYP52 family in yeast [32, 41] and the CYP4 family in mammals [48–51]. Therefore, CYP168A1 is unlikely to be involved in the production of α,ω-diacids. In order to hydroxylate fatty acids at the ω-carbon, CYP4B1 requires an unusual heme-polypeptide ester in the active site to mediate this energetically disfavored process [52].

As CYP168A1 does not catalyze the terminal ω-hydroxylation of fatty acids, it will not be involved in the production of α,ω-diacids. However, ω-1-fatty acids can be further converted to ω-1-oxo fatty acids. In *Legionella*, long chain ω-1-oxo fatty acids, also hydroxylated at the α-carbon (thus producing α-hydroxy, ω-1-oxo fatty acids), are constituents of the cell wall [53]. Also ω-1-oxo fatty acids can be further converted to ω-1-oxo dicarboxylic acids [54]. CYP168A1 does not metabolize sterols or steroids, which suggests the true substrates of CYP168A1 (if not fatty acids) are unlikely to be larger molecules. Alternatively, as in the case of CYP107H1, the fatty acids used in this study may reflect part of the true CYP168A1 substrate(s). Nevertheless, whilst the exact biological function of CYP168A1, like many other bacterial fatty acid hydroxylating CYPs, is yet to be determined, and requires further study. However, it can be hypothesized that CYP168A1 is involved in the degradation of fatty acids. Release of a high concentration of fatty acids (through high levels of phosphatidylcholine degradation) can be toxic to cells as they can cause the inhibition of enzymes involved in fatty acid degradation/β-oxidation [55, 56]. By hydroxylating these fatty acids through an enzyme, such as CYP168A1, the toxic effect can be lessened, and fatty acids can be stored.

It may be possible to use CYP168A1 as a potential target for novel anti-*Pseudomonas* drugs. Azole antifungals are a class of inhibitor that target CYP51 enzymes, presently optimized to inhibit fungal orthologs, through the direct coordination of the imidazole N-3 or the triazole N-4 nitrogen to the CYP51 heme ferric cation as the sixth axial ligand [19]. This mode of action also means that these azole antifungals are also able to bind to the heme iron of other CYPs, such as CYP52A21 from *Candida albicans* [41], CYP124 from *Mycobacterium tuberculosis* [40] and CYP164A2 from *M*. *smegmatis* [57].

CYP168A1 bound miconazole with greater affinity than CYP164A2 [57] and CYP124 [40], whilst CYP168A1 bound voriconazole with similar affinity to human CYP51 [58] and with greater affinity than *M*. *smegmatis* CYP51 [57]. However, CYP168A1 bound azole antifungals relatively poorly compared to fungal CYP51 enzymes, which typically gave *K*_d_ values of 0.004 to 0.05 μM [58–60]. When added to reconstitution assays, where oleic acid was used as the substrate, these azoles had no effect on the catalytic activity of CYP168A1 despite the azole concentration being eight times the concentration of the enzyme used, suggesting that once the substrate is bound to CYP168A1 it is not readily displaced by the azole antifungals investigated in this study. The azole ligand binding studies were performed using pure CYP168A1 enzyme in the absence of substrate and redox partners and that azole binding properties may differ in the CYP168A1 reconstitution assays. MIC experiments with the *P*. *aeruginosa* strains DSMZ 22644 and ATCC 39324, grown on oleic acid, agreed with this observation as tebuconazole, miconazole and voriconazole had no inhibitory effect on growth at concentrations up to 128 μg ml^-1^. By contrast, Tooker et al [28] reported that CYP168A1 was inhibited by ketoconazole when arachidonic acid was the substrate, resulting in a ∼70% reduction in enzyme activity with a CYP168A1:ketoconaozle ratio of 5μM:10μM, indicating screening a wider range of azole compounds may identify further CYP168A1 inhibitors. Currently available azole antifungal drugs would need to be redesigned and optimized to target CYP168A1 for such drugs to be considered effective inhibitors of *P*. *aeruginosa*.

This study has shown that the previously uncharacterized CYP168A1 from P. aeruginosa is involved in the sub-terminal hydroxylation of biologically relevant fatty acids. It is able to hydroxylate saturated fatty acids at both the ω-1- and ω-2-positions, but it is only able to hydroxylate unsaturated fatty acids at the ω-1-carbon.

## Supporting information

S1 Fig Mass fragmentation patterns of CYP168A1 assay metabolites. Mass fragmentation patterns for a) ω-1-hydroxy palmitic acid, diTMS, b) ω-2-hydroxy palmitic acid, diTMS, and c) ω-1-hydroxy oleic acid, diTMS are shown. TMS-derivatised fatty acids are identified by the [M-15]^+^ fragmentation ion.
(DOCX)
