# Supplementary material for: Cytochrome P450 168A1 from Pseudomonas aeruginosa is involved in the hydroxylation of biologically relevant fatty acids
Source: PLoS One. 2022 Mar 21;17(3):e0265227. doi: 10.1371/journal.pone.0265227 (PMC8936499; doi:10.1371/journal.pone.0265227)
Supplement: S1 Fig — Mass fragmentation patterns for a) ω-1-hydroxy palmitic acid, diTMS, b) ω-2-hydroxy palmitic acid, diTMS, and c) ω-1-hydroxy oleic acid, diTMS are shown. TMS-derivatised fatty acids are identified by the [M-15]+ fragmentation ion. (DOCX) [file pone.0265227.s001.docx]

Supplementary Information:

**Cytochrome P450 168A1 from *Pseudomonas aeruginosa* is involved in the hydroxylation of biologically relevant fatty acids**

Claire L. Price^1^, Andrew G. S. Warrilow^1^, Nicola J. Rolley^1^, Josie E. Parker^1^, Vera Thoss^2^, Diane E. Kelly^1^, Nicolae Corcionivoschi^3^, and Steven L. Kelly^1*^

^1^Centre for Cytochrome P450 Biodiversity, Institute of Life Science, Swansea University Medical School, Swansea University, Swansea, Wales SA2 8PP, United Kingdom

^2^Plant Chemistry Group, School of Chemistry, Bangor University, Bangor, Gwynedd, Wales LL57 2UW, United Kingdom

^3^Agri-Food and Biosciences Institute, Veterinary Science Division, Bacteriology Branch, Stoney Road, Stormont, Belfast, Northern Ireland BT4 3SD, United Kingdom


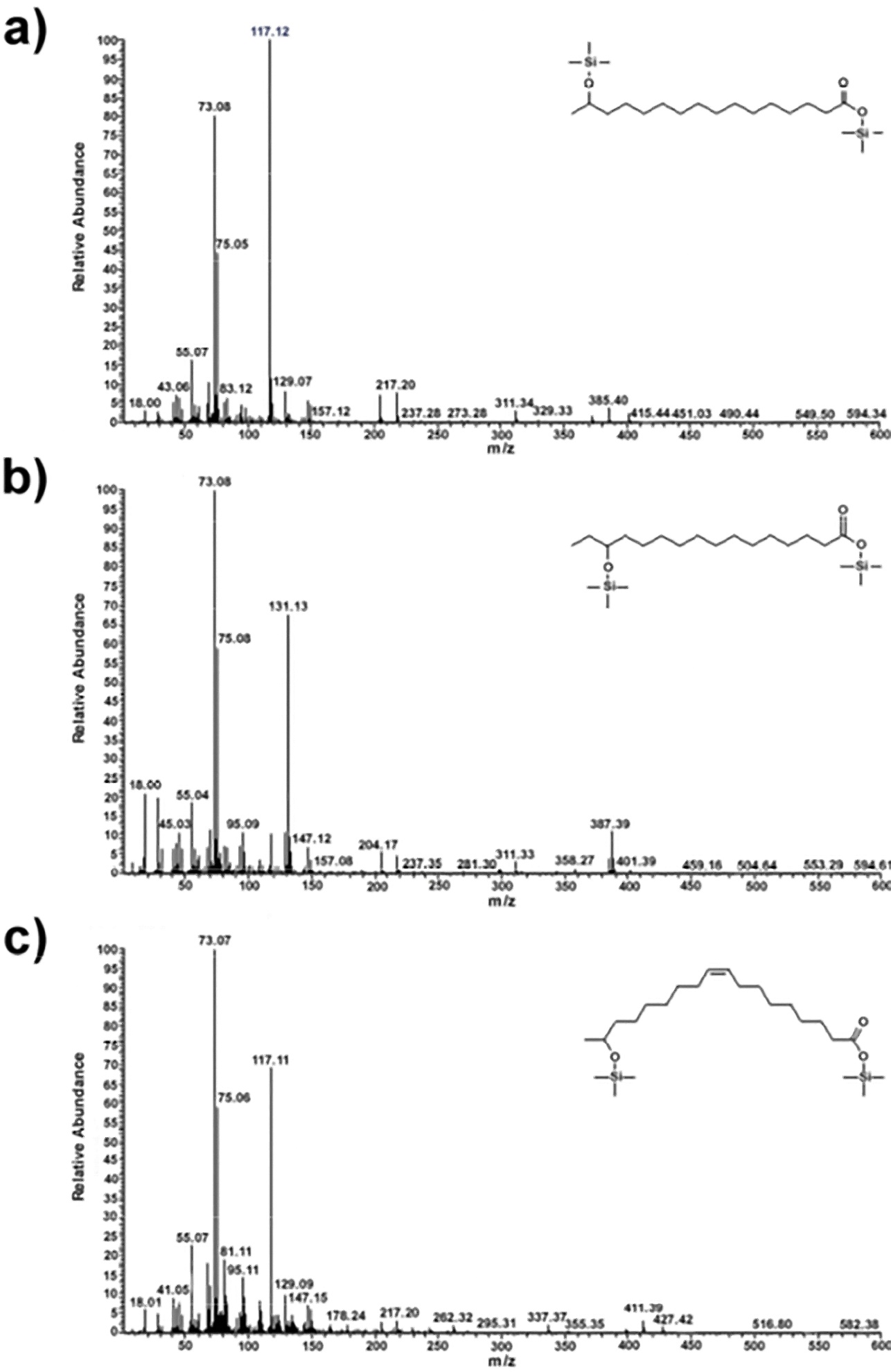


**Fig. S1** **Mass fragmentation patterns of CYP168A1 assay metabolites.** Mass fragmentation patterns for a) ω-1-hydroxy palmitic acid, diTMS, b) ω-2-hydroxy palmitic acid, diTMS, and c) ω-1-hydroxy oleic acid, diTMS are shown. TMS-derivatised fatty acids are identified by the [M-15]^+^ fragmentation ion.
